# Lifestyle intervention in obese pregnancy and cardiac remodelling in 3-year olds: children of the UPBEAT RCT

**DOI:** 10.1038/s41366-022-01210-3

**Published:** 2022-10-12

**Authors:** Paul D. Taylor, Haotian Gu, Hannah Saunders, Federico Fiori, Kathryn V. Dalrymple, Priyanka Sethupathi, Liana Yamanouchi, Faith Miller, Bethany Jones, Matias C. Vieira, Claire Singh, Annette Briley, Paul T. Seed, Dharmintra Pasupathy, Paramala J. Santosh, Alan M. Groves, Manish D. Sinha, Philip J. Chowienczyk, Lucilla Poston, UPBEAT Consortium, Lucilla Poston, Andrew Shennan, Annette Briley, Claire Singh, Paul Seed, Jane Sandall, Thomas Sanders, Nashita Patel, Angela Flynn, Shirlene Badger, Suzanne Barr, Bridget Holmes, Louise Goff, Clare Hunt, Judy Filmer, Jeni Fetherstone, Laura Scholtz, Hayley Tarft, Anna Lucas, Tsigerada Tekletdadik, Deborah Ricketts, Carolyn Gill, Alex Seroge Ignatian, Catherine Boylen, Funso Adegoke, Elodie Lawley, James Butler, Rahat Maitland, Matias Vieira, Dharmintra Pasupathy, Eugene Oteng-Ntim, Nina Khazaezadeh, Jill Demilew, Sile O’Connor, Yvonne Evans, Susan O’Donnell, Ari de la Llera, Georgina Gutzwiller, Linda Hagg, Stephen Robson, Ruth Bell, Louise Hayes, Tarja Kinnunen, Catherine McParlin, Nicola Miller, Alison Kimber, Jill Riches, Carly Allen, Claire Boag, Fiona Campbell, Andrea Fenn, Sarah Ritson, Alison Rennie, Robin Durkin, Gayle Gills, Roger Carr, Scott Nelson, Naveed Sattar, Therese McSorley, Hilary Alba, Kirsteen Paterson, Janet Johnston, Suzanne Clements, Maxine Fernon, Savannah Bett, Laura Rooney, Sinead Miller, Paul Welsh, Lynn Cherry, Melissa Whitworth, Natalie Patterson, Sarah Lee, Rachel Grimshaw, Christine Hughes, Jay Brown, Kim Hinshaw, Gillian Campbell, Joanne Knight, Diane Farrar, Vicky Jones, Gillian Butterfield, Jennifer Syson, Jennifer Eadle, Dawn Wood, Merane Todd, Asma Khalil, Deborah Brown, Paola Fernandez, Emma Cousins, Melody Smith, Jane Wardle, Helen Croker, Laura Broomfield, Weight Concern, Keith Godfrey, Sian Robinson, Sarah Canadine, Lynne Greenwood

**Affiliations:** 1 Department of Women & Children’s Health, School of Life Course Sciences, King’s College London, London, UK; 2 BHF Centre of Research Excellence, School of Cardiovascular Medicine and Sciences, King’s College London, London, UK; 3 Department of Child and Adolescent Psychiatry, IOPPN Kings College London, London, UK; 4 Reproduction and Perinatal Centre, Faculty of Medicine and Health, University of Sydney, Sydney, Australia; 5 Department of Pediatrics, Division of Newborn Medicine, Icahn School of Medicine at Mount Sinai, New York, NY USA; 6 Department of Paediatric Nephrology, Evelina London Children’s Hospital, King’s College London, Guy’s and St Thomas NHS Foundation Trust, London, UK; 7 King’s College London/Guy’s and St Thomas’ NHS Foundation Trust, London, UK; 8 King’s College Hospital, London, UK; 9 Newcastle University/Newcastle NHS Foundation Trust, Newcastle, UK; 10 Glasgow University and Greater Clyde Health board, Glasgow, UK; 11 Central Manchester Hospitals Foundation Trust, Manchester, UK; 12 City Hospital Sunderland, Sunderland, UK; 13 Bradford Royal Infirmary, Bradford, UK; 14 St George’s NHS Trust, London, UK; 15 University College London, London, UK; 16 University of Southampton, Southampton, UK

**Keywords:** Translational research, Risk factors, Paediatrics

## Abstract

**Background/Objectives:**

Obesity in pregnancy has been associated with increased childhood cardiometabolic risk and reduced life expectancy. The UK UPBEAT multicentre randomised control trial was a lifestyle intervention of diet and physical activity in pregnant women with obesity. We hypothesised that the 3-year-old children of women with obesity would have heightened cardiovascular risk compared to children of normal BMI women, and that the UPBEAT intervention would mitigate this risk.

**Subjects/Methods:**

Children were recruited from one UPBEAT trial centre. Cardiovascular measures included blood pressure, echocardiographic assessment of cardiac function and dimensions, carotid intima-media thickness and heart rate variability (HRV) by electrocardiogram.

**Results:**

Compared to offspring of normal BMI women (*n* = 51), children of women with obesity from the trial standard care arm (*n* = 39) had evidence of cardiac remodelling including increased interventricular septum (IVS; mean difference 0.04 cm; 95% CI: 0.018 to 0.067), posterior wall (PW; 0.03 cm; 0.006 to 0.062) and relative wall thicknesses (RWT; 0.03 cm; 0.01 to 0.05) following adjustment. Randomisation of women with obesity to the intervention arm (*n* = 31) prevented this cardiac remodelling (intervention effect; mean difference IVS −0.03 cm (−0.05 to −0.008); PW −0.03 cm (−0.05 to −0.01); RWT −0.02 cm (−0.04 to −0.005)). Children of women with obesity (standard care arm) compared to women of normal BMI also had elevated minimum heart rate (7 bpm; 1.41 to 13.34) evidence of early diastolic dysfunction (e prime) and increased sympathetic nerve activity index by HRV analysis.

**Conclusions:**

Maternal obesity was associated with left ventricular concentric remodelling in 3-year-old offspring. Absence of remodelling following the maternal intervention infers in utero origins of cardiac remodelling.

**Clinical trial registry name and registration number:**

The UPBEAT trial is registered with Current Controlled Trials, ISRCTN89971375.

## Introduction

Obesity amongst pregnant women has risen in parallel with population trends. In the UK more than half the women attending antenatal care are overweight or obese (BMI ≥25 kg/m^2^) [1]. Obesity increases the risk of perinatal and maternal morbidity and mortality, and is now the most prevalent risk factor for adverse pregnancy outcome [2], contributing to substantial healthcare costs [3]. Reports of an independent relationship between maternal BMI and body fat mass in their children have raised concern that maternal obesity may contribute directly to the global increase of obesity in childhood [4], prompting the suggestion that preventive strategies for reducing childhood obesity should include interventions targeting maternal BMI [5]. Cardiovascular risk in progeny of mothers with obesity, although less frequently investigated, includes a recent population-based cohort study demonstrating a greater risk of cardiovascular disease amongst 1–25-year-old offspring, which increased incrementally with the severity of maternal obesity, and another reporting an association between maternal obesity and all-cause mortality in adult offspring [6, 7]. Relationships between maternal BMI and childhood blood pressure have also been reported [8]. Using rodent models of maternal obesity, we and others, have demonstrated cardiac hypertrophy, hypertension and altered sympathetic activity in young offspring [9–12], implicating a direct effect of intrauterine exposures on cardiovascular development. In women and children, these associations could have other origins, including shared genetic traits and family environment [4]. Since randomised controlled trials (RCT) in pregnant women with obesity may offer insight into in utero versus familial origins of cardiovascular risk, this study evaluated the effect of a complex lifestyle intervention of diet and physical activity in obese pregnancy (the UPBEAT intervention) on childhood cardiovascular function.

The UK Pregnancies Better Eating and Activity Trial (UPBEAT) was a complex lifestyle intervention in 1555 pregnant women with obesity [13]. Women were randomised to an intense 8-week behavioural intervention or to standard antenatal care. The intervention had no effect on the primary outcomes, incidence of gestational diabetes and large for gestational age infants. However, there were improvements in several secondary maternal outcomes including; a reduction in total GWG and lower sum of skinfold thicknesses; an improvement in maternal antenatal glycaemic load and saturated fat intake; a modest increase in self-reported physical activity; a healthier metabolic profile across pregnancy [14]; as well as sustained improvements in maternal diet and lower infant adiposity (subscapular skinfold thicknesses) at 6 months postpartum [15]. The present study aimed to assess, in a subgroup of the original cohort, whether the UPBEAT intervention led to improved childhood cardiovascular function at 3 years.

The primary aims of this study were to compare detailed transthoracic echocardiographic measures in 3-year-old children from pregnant women with obesity with those from pregnant women of normal BMI (20–25 kg/m^2^). We hypothesised that children of women with obesity would have evidence of cardiovascular dysfunction compared to children of women with normal BMI, and that the UPBEAT intervention would mitigate this risk. Cardiovascular outcomes prioritised as risk factors for future cardiovascular disease included blood pressure, echocardiographic assessment of cardiac function (e.g., diastolic function) and dimensions (e.g., left ventricular mass (LVM)), and carotid intima-media thickness (CIMT) as a measure of vascular remodelling. Heart rate variability (HRV) by electrocardiogram (ECG) was also assessed as a marker of autonomic dysfunction, which we hypothesised may drive cardiovascular remodelling [16]. If persistent, these changes may contribute to adulthood cardiovascular disease.

## Methods

The UPBEAT trial was a multicentre RCT conducted on 1555 pregnant women with a median BMI of 35.1 kg/m^2^ (IQR 32.8–38.6) [13]. Maternal demographic details were obtained at trial entry. Women with obesity (≥16 years of age; pre-pregnancy BMI ≥30 kg/m^2^), were recruited in early pregnancy, exclusion criteria included pre-existing disease and multiple pregnancies. Women were randomised, at 15^+0^ and 18^+6^ weeks’ gestation, to an intensive 8-week behavioural intervention or to standard antenatal care, as previously reported [13]. In brief, the intervention comprised dietary recommendation to reduce maternal antenatal glycaemic load and saturated fat intake and to increase physical activity over an 8-week period. The intervention, delivered by health trainers, led to a healthier maternal metabolic profile, improved diet and lower weight gain in pregnancy and reduced infant adiposity at 6 months.

### Subjects

Mothers and their 3-year-old children attending the UPBEAT RCT follow-up visit at Guy’s and St. Thomas’ NHS Foundation Trust (GSTFT), London, UK, were informed about the additional nested case–control cardiovascular study and invited to return within 2 weeks, when the mother provided consent. The children were studied in the NIHR Clinical Research Facility at St. Thomas’ Hospital. Thirty-nine children were recruited from the standard care arm and 31 from the intervention arm from 228 mother–child pairs attending the 3-year-UPBEAT follow-up visit (Supplementary Fig. 1, Consort diagram).

Comparisons were also made between children from the standard care arm of the UPBEAT cohort, and 51 children of normal BMI mothers (20–25 kg/m^2^) who gave birth 3–4 years before the study date (i.e., contemporaneously with the UPBEAT cohort) who were identified from the hospital maternity database, GSTFT. The normal BMI women were matched for age and parity with women recruited to the UPBEAT standard care arm and recruited by letter.

### Consent and ethical approval

Informed consent was obtained from all subjects. Consent in the UPBEAT trial included an agreement for further contact (UK integrated research application system, reference 09/H0802/5). The follow-up study design and protocol were approved by the NHS Research Ethics Committee (UK Integrated Research Application System; reference 13/LO/1108). Research midwives and research assistants completed data collection between October 2014 and August 2018.

### Childhood outcomes

#### Echocardiography

A transthoracic echocardiographic study and the left common carotid artery image were obtained using the Philips Epiq ultrasound system (Philips Healthcare, Andover, USA) and analysed by one author (HG) blinded to the study group. All echocardiographic views and measurements were performed using standard techniques according to the American Society of Echocardiography [17–19] (see Supplementary Material for details).

#### Pulse wave velocity

Aortic pulse wave velocity (aPWV) was calculated using the time difference between R-wave to onset of pulsed wave Doppler at descending aorta (point of left subclavian artery) and abdominal aorta (point of diaphragm) using the length of the sternum between jugular notch and xiphoid process as a measure of path length.

#### Carotid intimal-medial thickness (IMT)

IMT was measured using the Philips QLAB vascular automated analysis package (Philips Healthcare, Andover, USA) focusing on the posterior wall over the left common carotid artery.

#### Blood pressure and heart rate variability

Blood pressure measurements were taken following 5 min of rest in a supine position, using the Welch Allyn 53S00-E4 device, with an appropriately sized arm cuff for the child. An average of three blood pressure readings were taken, and the mean, systolic and diastolic values were recorded. Blood pressure was subsequently converted to percentiles (adjusted for age, sex and height) using the Fourth report on the diagnosis, evaluation, and treatment of high blood pressure in children and adolescents [20, 21].

Following a further 5 min rest, the ECG was recorded over 20–30 min with the child in the supine position (ECG, lead II electrocardiogram, Medilog AR12 Plus). ECG traces were uploaded to the study database (MedSciNet) and analysed with Medilog Darwin 2 (ver. 2.7.1, SMART Medical, Gloucestershire, UK) and Kubios HRV Premium (ver. 3.3.1, MathWorks Inc. Massachusetts, United States) software [22, 23]. HRV was analysed from at least three 5-min segments of the recording obtained. Recordings were visually inspected, and non-sinusoidal beats and artefacts were excluded from analysis (see Supplementary Material).

### Statistics

Maternal and offspring characteristics in the form of numerical and categorical data were compared by *t*-test and *χ*^2^ test, respectively. Using unadjusted and adjusted regression analyses, comparisons were made between normal BMI controls (A) and UPBEAT standard care arms (C), and between UPBEAT intervention (B) and standard care (C) arms. Mean values with standard deviations were calculated for numerical data where appropriate. For the adjusted models, confounders included maternal ethnicity, maternal smoking status at UPBEAT trial baseline, age of the child at follow-up (months), sex and child BMI *z*-score. Analysis was by intention-to-treat. Analyses were performed using Stata version 15.0 (StataCorp, College Station, TX, USA) and IBM SPSS Statistics, Version 26.0. *P* ≤ 0.05 was considered significant (see Supplementary Material for sample size calculations).

Owing to the exploratory nature of this study, i.e., to examine childhood cardiovascular parameters potentially modifiable by the UPBEAT intervention in a subgroup of RCT participants, we did not control for multiple testing [24, 25]. This accords with reports of RCTs of exploratory and hypothesis-generating nature, which have adopted similar practice [26, 27].

## Results

### Subject characteristics

There were no differences in demographic or baseline variables between intervention and standard care arms in the subgroup of 70 women in the present study, as in the main UPBEAT trial (Table 1). Similarly, there was no difference in maternal fasting plasma glucose at 28 weeks gestation between intervention and standard care arms (Table 1). Although lower in the intervention arm, there was no statistical difference in the incidence of GDM between the intervention (23%) and standard care (30%) arms. Women in the normal BMI group, recruited contemporaneously from the general antenatal population were studied in parallel with the UPBEAT participants. UPBEAT participants were less likely to be white and to have a history of smoking than the normal BMI group, and had lower educational attainment, as assessed by years in education (Table 1).

**Table 1:** Maternal characteristics of normal BMI women (**A**), UPBEAT intervention arm (**B**), and UPBEAT standard care arm (**C**).

	Normal BMI (*n* = 52) (A)	UPBEAT intervention (*n* = 31) (B)	UPBEAT standard care (*n* = 39) (C)	*P* value A vs C	*P* value B vs C
*Maternal characteristics*					
Maternal age at delivery (years)	32.6 ± 4.40^a^	31.8 ± 5.37^a^	33.2 ± 5.40^a^	0.588	0.275
Ethnicity [*n* (%)]					
Asian	0 (0)	1 (3)	1 (2)	**0.024**^b^	0.464^b^
Black	14 (27)	11 (36)	19 (49)		
White	37 (71)	13 (42)	16 (41)		
Other	1 (2)	6 (19)	3 (7)		
BMI (kg/m^2^)	22.7 (20.9–23.6)	36.1 (33.8–38.9)	34.8 (32.3–37.7)	**<0.001**	0.286
Smoking status [*n* (%)]					
Current smoker	6 (11)	1 (3)	1 (3)	0.041^b^	0.817^b^
Quit before pregnancy	14 (27)	3 (10)	6 (15)		
Quit in pregnancy	0 (0)	4 (13)	3 (8)		
Never	32 (62)	23 (74)	29 (74)		
Number of previous pregnancies >37 weeks [*n* (%)]					
0	29 (56)	16 (52)	15 (38)	0.094^b^	0.302^b^
1	15 (27)	10 (32)	14 (36)		
2+	8 (15)	5 (16)	10 (26)		
Mode of delivery [*n* (%)]					
LSCS in labour	6 (12)	7 (23)	5 (13)	0.158^b^	0.675^b^
Operative vaginal	7 (13)	2 (6)	4 (10)		
LSCS prelabour	2 (4)	4 (13)	7 (18)		
Unassisted	37 (71)	18 (58)	23 (58)		
Years in education	17.17 ± 2.60^a^	14.97 ± 3.17^a^	14.69 ± 3.22^a^	**<0.001**	0.722
Maternal fasting plasma glucose 24–28 weeks pregnancy (mmol)	n/a^c^	4.60 ± 0.51^a^	4.77 ± 0.68^a^	n/a	0.240
GDM in pregnancy	n/a^c^	7 (23)	12 (30)		0.494
Maternal blood pressure at baseline					
Systolic	n/a^c^	116 ± 13.5^a^	116 ± 10.2^a^	n/a	0.941
Diastolic	n/a^c^	72.0 ± 10.1^a^	72.2 ± 7.2^a^		0.930

*IADPSG* International Association of Diabetes in Pregnancy Study Groups.

^a^Values are mean ± standard deviation unless specified as [*n* (%)].

^b^*P* values were calculated using independent samples *t*-test for continuous variables and *χ*^2^ tests for categorical variables.

^c^Data not available.

Bold values indicate significant *P* value (*P* < 0.05).

Comparing women in the UPBEAT subgroup to those in the original UPBEAT trial cohort [13, 14], maternal age was slightly higher by approximately 1 year, and ethnicity differed at baseline with more black and less Asian women represented at the London centre (Supplementary Table 1).

There was no difference in the sex ratio, birthweight, and gestational age at birth of offspring between the three study groups. All infants, except one, were born at term (>37 weeks gestation). The children in the normal maternal BMI group were slightly older at 3-year follow-up, by approximately 1 month, than the children in the UPBEAT standard care arm (*P* < 0.001) (Table 2).

**Table 2:** Characteristics of children of normal BMI women (**A**), UPBEAT intervention (**B**), and UPBEAT standard care arm (**C**) at birth.

	Normal BMI (*n* = 52) (A)	UPBEAT intervention (*n* = 31) (B)	UPBEAT standard care (*n* = 39) (C)	*P* value A vs C	*P* value B vs C
*Child characteristics*					
Sex [*n* (%)]					
Male	20 (38.5)	16 (51.6)	17 (43.6)	0.622	0.504
Female	32 (61.5)	15 (48.4)	22 (56.4)		
Birthweight (g)	3464 ± 421	3399 ± 562	3439 ± 482	0.790	0.750
Gestational age at birth (days)	282.33 ± 7.75	277.81 ± 7.38	278.96 ± 9.90	**0.04**	0.787
Age at follow-up (years)	3.88 ± 0.13	3.69 ± 0.20	3.74 ± 0.19	**<0.001**	0.257
Height-for-age *z*-score	0.17 ± 1.00	−0.31 ± 0.93	−0.31 ± 1.16	**0.149**	0.989

^a^Values are mean ± standard deviation unless specified as [*n* (%)].

^b^*P* values were calculated using independent samples *t*-test for continuous variables and *χ*^2^ tests for categorical variables.

Bold values indicate significant *P* value (*P* < 0.05).

### Obesity in pregnancy and child echocardiographic parameters

**Fig. 1:**
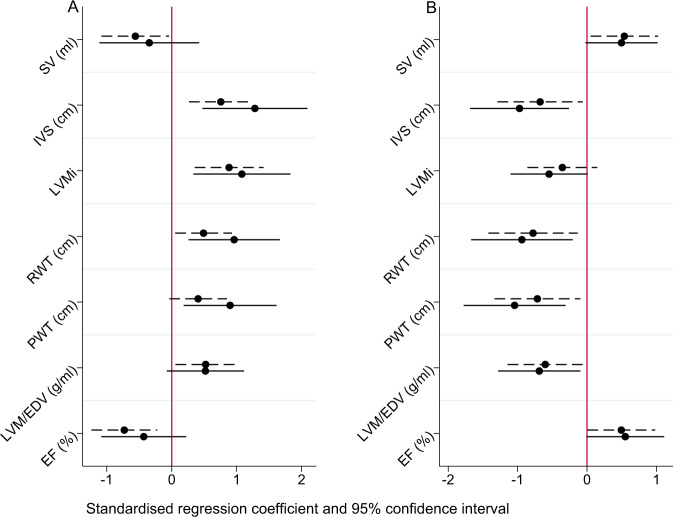
Summary of Echocardiography parameters for Left Ventricular Geometry and function. Echocardiographic parameters in children showing **A** maternal obesity effect and **B** intervention effect. Difference in standard deviation (SD), plotted with 95% confidence intervals are unadjusted (dash line) and adjusted (solid line). **A** Mean differences associated with the UPBEAT standard care arm (*n* = 39) versus the normal BMI group [51] are shown on the left and **B** mean differences associated with the UPBEAT intervention arm (*n* = 31) versus the UPBEAT standard care arm (*n* = 39) are shown on the right.

Compared to children of women with a normal BMI, children in the UPBEAT standard care arm demonstrated increased interventricular septal thickness (IVS) thickness after adjustment for maternal ethnicity and smoking status at baseline, child age, gender and BMI *z*-score (IVS, mean diff (CI): 0.04 cm; 0.018 to 0.067); they also demonstrated increased posterior wall thickness (PWT, 0.03 cm (0.006 to 0.062) and relative wall thickness after adjustment (RWT 0.03 cm; 0.01 to 0.054). Evidence of LV concentric remodelling included higher LVM index (LVMi g/m^2^; 3.96; 1.36 to 6.57), and an increase in the ratio of LVM to end-diastolic volume (LVM/EDV; 0.95 g/ml; 0.01 to 0.17, Table 3 and Fig. 1).

**Table 3:** Echocardiographic measurements of children of normal BMI women (**A**), UPBEAT intervention (**B**), and UPBEAT standard care arm.

	Normal BMI (*n* = 51)	UPBEAT intervention (*n* = 30)	UPBEAT standard care (*n* = 39)	Obesity effect, mean diff (CI)	Unadjusted *P* value	Adjusted obesity effect	Adjusted *P* value	Intervention effect, mean diff (CI)	Unadjusted *P* value	Adjusted intervention effect	Adjusted *P* value
*LV geometry*											
EDV (ml)	31.8 ± 4.4	33.2 ± 6.5	30.8 ± 4.8	−0.95 (−2.87 to 0.97)	0.331	−0.41 (−2.64 to 1.83)	0.717	2.41 (−0.30 to 5.13)	0.081	1.66 (−1.04 to 4.37)	0.223
ESV (ml)	10.8 ± 2.2	11.4 ± 2.6	11.3 ± 2.2	0.50 (−0.43 to 1.44)	0.287	0.22 (−0.89 to 1.33)	0.69	0.07 (−1.11 to 1.25)	0.907	−0.17 (−1.36 to 1.01)	0.774
SV (ml)	20.9 ± 2.8	21.8 ± 4.8	19.5 ± 3.4	**−1.45 (−2.76 to −0.14)**	**0.030**	−0.63 (−2.17 to 0.90)	0.416	**2.34 (0.35 to 4.33)**	**0.021**	1.83 (−0.16 to 3.84)	0.072
CO (l/min)	2.11 ± 0.36	2.20 ± 0.45	2.03 ± 0.41	−0.08 (−0.24 to 0.08)	0.338	0.07 (−0.11 to 0.26)	0.446	0.16 (−0.04 to 0.37)	0.112	0.11 (−0.10 to 0.32)	0.319
EDD (cm)	3.18 ± 0.26	3.20 ± 0.23	3.14 ± 0.24	−0.04 (−0.14 to 0.07)	0.474	−0.08 (−0.21 to 0.05)	0.236	0.06 (−0.05 to 0.17)	0.324	0.03 (−0.08 to 0.15)	0.607
ESD (cm)	2.05 ± 0.22	2.02 ± 0.21	2.02 ± 0.21	−0.01 (−0.11 to 0.07)	0.698	−0.04 (−0.15 to 0.06)	0.440	−0.006 (−0.10 to 0.09)	0.899	−0.03 (−0.12 to 0.06)	0.461
IVS (cm)	0.44 ± 0.06	0.45 ± 0.05	0.47 ± 0.04	**0.03 (0.01 to 0.05)**	**<0.001**	**0.04 (0.018 to 0.067)**	**0.001**	**−0.02 (−0.04 to −0.002)**	**0.026**	**−0.03 (−0.05 to −0.008)**	**0.008**
PW (cm)	0.46 ± 0.06	0.45 ± 0.04	0.48 ± 0.05	0.02 (−0.001 to 0.04)	0.07	**0.03 (0.006 to 0.062)**	**0.016**	-**0.02 (−0.05 to −0.005)**	**0.019**	**−0.03 (−0.05 to −0.01)**	**0.005**
RWT	0.28 ± 0.05	0.28 ± 0.03	0.30 ± 0.04	**0.02 (0.002 to 0.04)**	**0.024**	**0.03 (0.01 to 0.054)**	**0.005**	**−0.02 (−0.04 to −0.005)**	**0.013**	**−0.02 (−0.04 to −0.005)**	**0.012**
LVM (g)	30.3 ± 5.1	31.0 ± 5.6	32.0 ± 4.9	1.68 (−0.44 to 3.80)	0.118	1.90 (−0.74 to 4.55)	0.156	−0.99 (−3.50 to 1.52)	0.434	−1.90 (−4.42 to 0.60)	0.134
LVMi (g/m^2.7^)	28.4 ± 4.2	30.7 ± 4.5	32.4 ± 5.5	**3.89 (1.86 to 5.92)**	**<0.001**	**3.96 (1.36 to 6.57)**	**0.003**	−1.73 (−4.20 to 0.73)	0.163	−2.45 (−5.06 to 0.159)	0.065
LVM/EDV (g/ml)	0.96 ± 0.18	0.95 ± 0.18	1.06 ± 0.20	**0.95 (0.01 to 0.17)**	**0.022**	0.08 (−0.02 to 0.18)	0.127	−0.11 (−0.20 to −0.014)	**0.024**	**−0.11 (−0.21 to −0.012)**	**0.028**
*LV function*											
EF (%)	66.0 ± 4.0	65.6 ± 4.9	63.1 ± 4.9	**−2.89 (−4.75 to −1.04)**	**0.003**	−1.36 (−3.61 to 0.88)	0.231	**2.45 (0.09 to 4.81)**	**0.042**	2.30 (−0.13 to 4.74)	0.063
FS (%)	35.4 ± 7.0	36.7 ± 4.6	35.3 ± 5.8	−0.12 (−2.88 to 2.64)	0.931	−0.22 (−3.76 to 3.32)	0.901	1.46 (−1.12 to 4.06)	0.263	1.83 (−0.78 to 4.45)	0.165
GLS (%)	−18.0 ± 2.2	−18.1 ± 1.9	−17.6 ± 2.0	0.46 (−0.52 to 1.46)	0.350	0.86 (−0.37 to 2.10)	0.167	−0.53 (−1.55 to 0.50)	0.302	−0.98 (−2.12 to 0.14)	0.085
S (m/s)	0.08 ± 0.01	0.09 ± 0.01	0.08 ± 0.01	−0.002 (−0.007 to 0.003)	0.410	−0.003 (−0.01 to 0.004)	0.364	0.005 (−0.0003 to 0.01)	0.066	0.005 (−0.001 to 0.012)	0.102
e’ (m/s)	0.15 ± 0.03	0.15 ± 0.02	0.14 ± 0.02	−0.008 (−0.017 to 0.001)	0.066	**−0.08 (−0.14 to −0.02)**	**0.01**	0.004 (−0.005 to 0.01)	0.352	0.02 (−0.044 to 1.01)	0.441
a’ (m/s)	0.068 ± 0.02	0.068 ± 0.01	0.074 ± 0.02	0.006 (−0.002 to 0.015)	0.126	0.03 (−0.02 to 0.10)	0.257	−0.005 (−0.01 to 0.004)	0.238	−0.03 (−0.107 to 0.041)	0.380
E/A	1.83 ± 0.50	1.78 ± 0.49	1.58 ± 0.35	**−0.23 (−0.42 to −0.05)**	**0.013**	**−0.24 (−0.47 to −0.0005)**	**0.050**	0.18 (−0.013 to 0.39)	0.067	0.18 (−0.036 to 0.40)	0.100
E/e’	6.7 ± 1.4	6.9 ± 1.8	6.8 ± 1.3	0.15 (−0.41 to 0.72)	0.597	−0.27 (−0.98 to 0.43)	0.443	0.069 (−0.67 to 0.81)	0.853	0.14 (−0.63 to 0.91)	0.713
LAV (ml)	13.5 ± 3.1	14.8 ± 4.3	15.6 ± 4.4	**2.13 (0.56 to 3.70)**	**0.008**	1.46 (−0.52 to 3.45)	0.146	−0.84 (−2.95 to 1.26)	0.428	−1.02 (−3.27 to 1.20)	0.362
LAVi (ml/m^2^)	19.5 ± 4.3	21.7 ± 6.1	23.1 ± 6.1	**3.54 (1.35 to 5.73)**	**0.002**	2.77 (−0.06 to 5.54)	0.051	−1.43 (−4.40 to 1.54)	0.340	−1.55 (−4.83 to 1.71)	0.344
*Aortic stiffness*											
PWV (m/s)	4.0 ± 1.1	4.6 ± 2.1	4.3 ± 1.5	0.25 (−0.31 to 0.82)	0.370	0.43 (−0.31 to 1.18)	0.253	0.29 (−0.63 to 1.22)	0.533	0.32 (−0.67 to 1.32)	0.519
CIMT (mm)	0.46 ± 0.04	0.47 ± 0.04	0.47 ± 0.04	0.01 (−0.005 to 0.03)	0.160	0.01 (−0.015 to 0.036)	0.417	−0.0001 (−0.02 to 0.02)	0.987	0.005 (−0.17 to 0.027)	0.635

Values are mean ± standard deviation. Una*P* values were calculated using regression analysis. A*P* values were calculated using regression analysis with adjustment for maternal ethnicity and smoking status at baseline, child age at follow-up, gender and BMI *z*-score at follow-up.

*EDV* end-diastolic volume, *ESV* end-systolic volume, *SV* stroke volume, *CO* cardiac output, *EDD* end-diastolic diameter, *ESD* end-systolic diameter, *IVS* interventricular septal thickness, *PW* posterior wall thickness, *RWT* relative cardiac wall thickness, *LVM* left ventricular mass, *LVMi* left ventricular mass index, *EF* ejection fraction, *FS* fractional shortening, *GLS* global longitudinal strain, *S* S wave, *e’* e prime wave, *a’* a prime wave, *E* E wave (peak velocity blood flow from left ventricular relaxation in early diastole), *A* A wave (peak velocity flow in late diastole caused by atrial contraction), *LAV* left atrial volume, *LAVi* left atrial volume index (height × height), *PWV* aortic pulse wave velocity, *CIMT* carotid artery intermedia thickness.

Bold values indicate significant *P* value (*P* < 0.05).

Functional measures suggested early indications of diastolic dysfunction in children in the UPBEAT standard care arm compared to children of women with a normal BMI, as evidenced by a preserved and lower E/A ratio (E/A, −0.23 (−0.42 to −0.05)) that remained significant after adjustment (E/A, −0.24 (−0.47 to −0.0005). A reduction in e prime, also significant after adjustment (e’, −0.08 m/s; −0.14 to −0.02) suggested slower early diastolic filling (Table 3).

### UPBEAT intervention in pregnancy prevents LV concentric remodelling

Compared to children of women in the UPBEAT standard care arm, those in the intervention arm did not show evidence of concentric remodelling; rather there was a significant reduction in IVS thickness (−0.02 cm; −0.04 to −0.002), a reduction in PW thickness (−0.02 cm; −0.05 to −0.005), RWT (−0.02; −0.04 to −0.005), and in the ratio of LVM to EDV (−0.11 g/ml; −0.20 to −0.014), all of which remained significant after adjustment (Fig. 1 and Table 3).

### Pulse wave velocity and carotid intimal-medial thickness (CIMT)

Across all groups, maternal BMI was not related to offspring PWV or CIMT and there was no apparent effect of the intervention (Table 3).

### Heart rate and autonomic function

Children in the UPBEAT standard care arm had significantly increased minimum heart rate (min HR; 7 bpm; 1.4 to 13.3), maximum HR (8 bpm; 0.94 to 15.1) and mean HR (6 bpm; 1.0 to 12.5) compared to children of normal BMI mothers, after adjustment for maternal ethnicity and smoking status at baseline, child age at follow-up, gender and BMI *z*-score at follow-up (Table 4).

**Table 4:** HR and HRV analysis of control women of normal weight (**A**), UPBEAT intervention (**B**), and UPBEAT standard care arm (**C**).

	Normal BMI (*n* = 51)	UPBEAT intervention (*n* = 31)	UPBEAT standard care (*n* = 39)	Obesity effect, mean diff (CI)	*P* value	Obesity effect adjusted, mean diff (CI)	Adjusted *P* value	Intervention effect, mean diff (CI)	*P* value	Intervention effect adjusted, mean diff (CI)	Adjusted *P* value
Mean HR (bpm)	102 ± 9.4	106 ± 9.4	107 ± 11.3	**4.56 (0.17 to 8.96)**	**0.04**	**6.76 (1.03 to 12.50)**	**0.021**	−0.30 (−5.44 to 4.83)	0.90	−0.05 (−5.60 to 5.50)	0.985
Min HR (bpm)	84 ± 10.2	87 ± 10.2	90 ± 10.8	**5.45 (0.93 to 9.96)**	**0.018**	**7.38 (1.41 to 13.34)**	**0.016**	−0.91 (−5.96 to 4.13)	0.71	−0.94 (−6.21 to 4.31)	0.720
Max HR (bpm)	126 ± 11.4	129 ± 9.0	132 ± 14.0	5.05 (−0.34 to 10.4)	0.06	**8.01 (0.94 to 15.1)**	**0.027**	−2.12 (−8.01 to 3.76)	0.474	−2.06 (−8.33 to 4.21)	0.513
SDNN (ms)	36.4 ± 11.7	32.8 ± 10.5	32.8 ± 10.8	−3.62 (−8.49 to 1.25)	0.143	**−7.13 (−13.30 to −0.95)**	**0.024**	0.063 (−5.15 to 5.28)	0.981	1.03 (−4.39 to 6.46)	0.704
RMSSD (ms)	36.3 ± 16.0	32.0 ± 13.5	32.7 ± 15.1	−3.60 (−10.31 to 3.09)	0.288	**−8.97 (−17.3 to −0.65)**	**0.035**	−0.70 (−7.74 to 6.33)	0.842	0.55 (−6.7 to 7.86)	0.879
pNN50 (%)	17.0 ± 14.0	13.7 ± 22.8	14.3 ± 12.7	−2.71 (−8.50 to 3.07)	0.354	**−7.41 (−14.4 to −0.35)**	**0.04**	−1.18 (−7.22 to 4.85)	0.696	0.10 (−6.00 to 6.22)	0.975
LF power (%)	48.6 ± 11.2	51.1 ± 11.1	45.6 ± 12.8	−3.05 (−8.16 to 2.05)	0.238	−0.39 (−6.90 to 6.11)	0.905	5.50 (−0.40 to 11.4)	0.067	4.38 (−1.83 to 10.6)	0.163
HF power (%)	42.9 ± 13.0	40.6 ± 12.8	46.4 ± 15.4	3.55 (−2.48 to 9.59)	0.245	0.46 (−7.19 to 8.11)	0.905	−5.79 (−12.7 to 1.19)	0.103	−4.35 (−11.6 to 2.90)	0.235
LF/HF	1.4 ± 0.88	1.4 ± 0.73	1.2 ± 0.74	−0.15 (−0.50 to 0.19)	0.386	−0.01 (−0.47 to 0.44)	0.946	0.24 (−0.11 to 0.61)	0.17	0.18 (−0.20 to 0.57)	0.336
SD1 (ms)	25.7 ± 11.4	22.6 ± 9.6	23.1 ± 10.6	−2.55 (−7.29 to −2.18)	0.288	**−6.34 (−12.2 to −0.45)**	**0.035**	−0.50 (−5.48 to 4.47)	0.841	0.38 (−4.77 to 5.56)	0.881
SD2 (ms)	44.4 ± 13.0	40.4 ± 12.1	39.9 ± 11.9	−4.47 (−0.83 to 0.89)	0.101	**−7.94 (−14.81 to −1.07)**	**0.024**	0.44 (−5.42 to 6.32)	0.880	1.52 (−4.57 to 7.62)	0.619
SD2/SD1	1.89 ± 0.46	1.92 ± 0.46	1.91 ± 0.52	0.02 (−0.18 to 0.23)	0.835	0.16 (−0.90 to 0.43)	0.197	0.014 (−0.23 to 0.25)	0.906	−0.009 (−0.27 to 0.25)	0.939
PNS	−1.7 ± 0.75	−1.9 ± 0.68	−1.9 ± 0.78	−0.24 (−0.57 to 0.08)	0.141	**−0.48 (−0.90 to −0.06)**	**0.024**	−0.02 (−0.38 to 0.34)	0.916	0.009 (−0.38 to 0.39)	0.962
SNS	3.14 ± 1.32	3.6 ± 1.49	3.73 ± 1.51	0.59 (−0.009 to 1.19)	0.054	**0.98 (0.19 to 1.76)**	**0.015**	−0.07 (−0.81 to 0.65)	0.831	−0.07 (−0.85 to 0.70)	0.857

Data presented as mean (SD) or median (IQR) as appropriate.

*SDNN* standard deviation of the normal to normal beat variation, *RMSSD* root mean square of standard deviation of the normal to normal beat variation, *LF* low-frequency component of HRV, *HF* high-frequency component of HRV, *SD1* standard deviation 1 and *SD2* from Poincare plots, where SD1 and SD2 are the standard deviations perpendicular to and along the line-of-identity (RRn = RRn + 1), *PNS* index of parasympathetic nervous system activity, *SNS* index of sympathetic nervous system activity.

Adjusted *P* values were calculated using regression analysis with adjustment for maternal ethnicity and smoking status at baseline, child age at follow-up, gender, and BMI *z*-score at follow-up.

Bold values indicate significant *P* value (*P* < 0.05).

Time domain analysis revealed a decrease in the standard deviation of the normal to normal beat variation (−7.13 ms; −13.3 to −0.95) indicating reduced HRV; a reduction in the root mean square of successive differences (−8.9; −17.3 to −0.65) and the proportion of successive NN intervals that differ by more than 50 ms (pNN50, −7.41; −14.4 to −0.35) suggest reduced parasympathetic activity in the UPBEAT standard care arm compared to the normal BMI group, after adjustment (Table 4).

Children in the UPBEAT standard care arm showed an increase in sympathetic nervous system (SNS) activity and a reduction in parasympathetic nervous system (PNS) activity relative to the normal BMI group, after adjustment (SNS index: 0.98; 0.19 to 1.76: PNS index: −0.48; −0.90 to −0.06, Table 4).

Whilst there was a trend towards improvement of all parameters of HR and HRV in the UPBEAT intervention arm compared to the UPBEAT standard care arm, these differences were not significant.

### Maternal obesity in pregnancy and child blood pressure

There was no association between maternal BMI and child systolic or diastolic blood pressure percentiles across the groups (Table 5).

**Table 5:** Blood pressure percentiles in children of normal BMI women (**A**), UPBEAT intervention (**B**), and UPBEAT standard care arm.

	*n*	Normal BMI (*n* = 46) (A)	*n*	UPBEAT intervention (*n* = 31) (B)	*n*	UPBEAT standard care (*n* = 39) (C)	Model 1: unadjusted		Model 2: adjusted	
							*P* value A vs C	*P* value B vs C	*P* value A vs C	*P* value B vs C
Average systolic BP (mmHg)	46	72 (61–82)	31	83 (68–88)	39	78 (61–82)	0.181	0.359	0.19	0.29
Average diastolic BP (mmHg)	46	64 (50–83)	31	75 (55–85)	39	70 (53–83)	0.444	0.515	0.28	0.89

Values are median (IQR). *P* values were calculated using unadjusted and adjusted quantile regression (confounders: maternal smoking status at baseline, maternal ethnicity, and child’s BMI *z*-score).

## Discussion

### Children born to women with obesity in pregnancy versus women of normal BMI

Despite population-based evidence for increased cardiovascular risk in adults born to women with obesity [7], few investigators have addressed associations between maternal obesity and childhood cardiovascular outcomes [8, 28–30], with only one reporting on children of pre-school age [31]. One has addressed the effect of a prenatal intervention with metformin [32]. Our observation that children born to women with obesity have an elevated heart rate and evidence for concentric remodelling of the heart, independent of childhood BMI, contrasts with observations in older children from Generation R, a large cohort in the Netherlands in which associations between maternal BMI and LVM, LVMi and concentric LVH in the 6-year-old children became non-significant after adjustment for child’s BMI [33]. However, most women in that study were of normal BMI, only 8% being obese.

Although we found that some differences observed in left ventricular function (LVM/EDV ratio and SV, EF, LAV and LAVi) between children of women with obesity versus mothers of normal BMI did not persist after adjustment (including childhood BMI) others were maintained, including a slower E wave and E/A ratio, suggesting that the LV concentric remodelling observed was associated with slower LV filling, and an early indication of diastolic dysfunction, respectively.

Our study also contrasts with Litwin et al. [30] who, in a cohort of older children of women with obesity from the Finnish Gestational Diabetes Prevention Study (RADIEL), found that LVM, LVMi and LVM *z*-score were not associated with pre-pregnancy BMI, or gestational diabetes, or child body fat percentage, but rather with lean body mass at 6 years of age [30]. Whilst we found no apparent difference in BP or CIMT in our 3-year olds born to women with obesity, the RADIEL study also reported increased BP and increased carotid intima thickness in 6-year-old children of mothers with obesity compared to a reference population [8]. Arterial dimensions were again mainly predicted by child lean body mass, and not associated with maternal or child adiposity, or GDM [8].

### Effects of a lifestyle intervention in women with obesity in pregnancy on offspring cardiovascular development

The association between maternal obesity and childhood cardiac structure and function might be attributed to common genetic cardiovascular traits in mother and child or to a shared family environment. However, this and other recent studies might argue in favour of a direct influence of maternal obesity during critical windows of development in utero. We report here that the UPBEAT lifestyle intervention in pregnancy complicated by obesity, attenuated the observed cardiac remodelling in the children with some improvement in cardiac function. As this was a representative subgroup from a well-conducted randomised control trial this should mitigate against confounding variables. We cannot discount a persisting influence of the UPBEAT pregnancy lifestyle intervention on mothers, influencing the family environment and childhood cardiovascular risk. However, data from the UPBEAT 6-month and 3-year follow-ups suggest no difference in maternal infant feeding practices [34] or the child’s dietary patterns and/or eating behaviours at 3 years of age [35]. Moreover, in the current UPBEAT subgroup, there were no differences in the mode of infant feeding on hospital discharge, mode of feeding at 6 months of age, or dietary patterns at 3 years of age between randomisation groups.

A direct in utero effect of maternal obesity becomes more likely in the context of parallel observations in foetuses and neonates of women with obesity. Abnormalities in the cardiac structure of offspring of women with obesity have been reported as early as 14 weeks’ gestation and are consistent with animal studies of maternal obesity-induced fibrosis in fetus myocardium [36]. Previous studies have reported increased foetal LV and RV global strain at 14 weeks’ gestation in women with obesity, compared to lean mothers, as assessed by ultrasound, a defect that persisted at 20 and 32 weeks’ gestation [36] and persists after birth. Moreover, compared to offspring of women of normal BMI, newborns of women with obesity have been shown to have larger and thicker left ventricles and an increased SV and EDV at 12 months of age [37]. Our study would suggest that these subclinical changes persist into pre-school years and there is evidence they may well track through childhood [38, 39]. Subclinical structural changes in children may be predictive of future cardiovascular events in adulthood [40].

### Potential role of autonomic dysfunction in infant cardiac remodelling

In association with increased LVM and cardiac wall thickening, we also report altered autonomic activity in the 3-year olds as evidenced by increased heart rate in the children of women with obesity vs women of normal BMI and an increase in SNS index and a decrease in PNS index of the HRV. Although in this subgroup there was no significant effect of the UPBEAT intervention on the children’s heart rate, we have observed in the follow-up study of 514 UPBEAT 3-year olds a lower resting pulse rate (−5 bpm; −8.41, −1.07) in children from the UPBEAT intervention arm versus those from the UPBEAT standard care arm [35]. Several studies also report a higher heart rate and/or reduced HRV in foetuses of women with obesity [16, 41, 42].

These observations of altered autonomic nervous system function are paralleled in experimental animals. In young offspring of obese rodents, we have found, in association with hypertension, a shift in the sympathetic to parasympathetic ratio of HRV, indicative of heightened sympathetic efferent tone [43, 44] which persists to adulthood, and is mimicked by exposure to hyperleptinemia in new-born pups [43]. Hence, we have proposed that hyperleptinemia exposure, at the level of the developing hypothalamus, may play a permissive role in increased sympathetic efferent activity, leading to offspring cardiac dysfunction and hypertrophy.

Although animal models have provided some insight into potential mechanisms, any extrapolation to the human condition of maternal obesity must remain conjectural. An absence of differences in cord blood leptin in the original UPBEAT cohort (*n* = 698) between intervention and standard care arms would not support the leptin hypothesis [34]. Although the UPBEAT subgroup did not demonstrate significant differences in HR or parameters of HRV between randomisation arms, despite trends towards improvements, as mentioned above the larger cohort did demonstrate a significant reduction in resting pulse rate in the intervention group [35]. This could infer a role for sympathetic hyperactivity in the cardiac dysfunction observed, and its prevention in the intervention arm.

Several reports propose that maternal insulin resistance and/or diabetes may play a role in both altered foetal and neonatal HRV and offspring LVH [45–47] but we found no difference in the incidence of gestational diabetes or in fasting glucose at the time of the oral glucose tolerance test (24–28 weeks’ gestation) in pregnant women with obesity between standard care and intervention arms, suggesting independence from glucose status in the differences observed.

Other potential mediators of the effect of maternal obesity on cardiac remodelling, that may be mitigated by the UPBEAT intervention include lipotoxity in the placenta [48], leading to placental inflammation, oxidative stress, cardiac inflammation [49] and foetal myocyte hypertrophy [50].

## Conclusions

Maternal obesity was associated with LV concentric remodelling in 3-year-old offspring that was prevented by the UPBEAT diet and exercise intervention in pregnancy suggesting the in utero developmental origins of cardiac remodelling and implicating an underlying elevated heart rate and increased sympathetic drive in the aetiology. Despite the relatively small cohort size in this exploratory study, the principal strength lies in the detailed phenotyping of the mother and children in the UPBEAT trial, and the breadth of cardiovascular measures undertaken in the child.

Subclinical cardiac hypertrophy in pre-school-age children secondary to maternal obesity may be a surrogate endpoint for the prediction of future cardiovascular risk [51] but could also be an antecedent to hypertension and cardiovascular disease [52, 53]. Evaluation of the same parameters in longitudinal prospective studies of cardiovascular risk in young people would be of interest. A recent systematic review identified consistent evidence of associations between maternal obesity and offspring cardiovascular dysfunction throughout the lifecourse, supporting targeted maternal obesity interventions for the promotion of offspring cardiovascular health [54].

A possible limitation is the sample size of those eligible from the original RCT, which may have resulted in selection bias, apparent in some minor differences by randomised group in maternal characteristics between those lost to follow-up and those included in the analyses (Supplementary Table 1). The unique findings of the effect of a pregnancy intervention (RCT) on the long-term cardiovascular health of the progeny warrant follow-up of the children in all the UPBEAT trial centres as they grow to maturity and funding has now been secured from the British Heart Foundation for a full cardiovascular assessment of the UPBEAT children at 10 years of age.

## Supplementary information

Supplementary material

## Data Availability

All data generated or analysed during this study are included in this published article [and its Supplementary information files]. Data collected for this study, including individual participant data and a data dictionary defining each field in the set, will be made available to others, upon request following publication. Proposals to use data from the UPBEAT RCT are considered by the UPBEAT Scientific Committee. In the first instance, scientists interested in using these data should contact the UPBEAT principal investigator LP at lucilla.poston@kcl.ac.uk.
