# Supplementary material for: Lifestyle intervention in obese pregnancy and cardiac remodelling in 3-year olds: children of the UPBEAT RCT
Source: Int J Obes (Lond). 2022 Oct 12;46(12):2145–55. doi: 10.1038/s41366-022-01210-3 (PMC9678793; doi:10.1038/s41366-022-01210-3)
Supplement: Supplementary file 1 — Supplementary material [file 41366_2022_1210_MOESM1_ESM.doc]

**SUPPLEMENTAL MATERIAL**

**Lifestyle Intervention in Obese Pregnancy and Cardiac Remodelling in 3-year-olds: Children of the UPBEAT RCT**

**Taylor, ‘Maternal Obesity and Cardiac Remodelling in Children’**

**Supplemental Methods**

***Sample size calculation:*** The original Sample size calculation for the study was based on 37 children in each of the UPBEAT control and intervention arms, and 37 children of normal BMI control women. This was based on the the values for infant left ventricular mass (LVM) ^1^, LVM/height^2^ has median 25.8 g/m^2^, 95th centile 33.6; implying a SD of 4.74. Full data on 37 three-year-old infants in each experimental arm would have provided 80% power to detect a significant 12% mean difference in LVM (25.8 to 22.7 g/m^3^).

Despite the cohort size, retrospective sample size calculations based on the data obtained for infant LVMi, with a mean of 28·4 ±4·2 g/m^2^ for the normal weight controls suggested 26 children in each of the UPBEAT control and intervention arms were sufficient to provide 80% power to detect a significant 15% difference in mean for LVMi (28·4 to 32·7 g/m^2^), and that the study was therefore adequately powered.

***Inclusion criteria:*** Children of women recruited to UPBEAT RCT aged 3-4-years-old; children of women with normal BMI matched for maternal age at the time of pregnancy to the women recruited from the standard care arm (*Supplemental Fig. 1*).

***Exclusion Criteria*** All women, inability / unwillingness to provide informed consent. For women of normal BMI, multiple pregnancy, pre-existing medical history at the time of the index pregnancy (GDM, diabetes, hypertension, renal disease, lupus, and antiphospholipid syndrome). All children: birth <34 weeks’ gestation, severe illness which could affect growth or development.

***Echocardiography*** A transthoracic echocardiographic study and the left common carotid artery image were obtained using the Philips Epiq ultrasound system (Philips Healthcare, Andover, USA) and analysed by one author (HG) blinded to study group. All echocardiographic views and measurements were performed using standard techniques according to American Society of Echocardiography (ASE)^2^. LVM was measured by two-dimensional directed M-mode echocardiography according to ASE guidelines ^2^, and indexed to height and (expressed as g/m^2.7^) ^3^. Left ventricular end-diastolic diameter (EDD), interventricular septal (IVS) and posterior wall (PW) thickness were measured from a parasternal long axis view. Relative wall thickness (RWT) was calculated using average of IVS and PW thicknesses divided by EDD. LV volumes and ejection fraction (EF) were derived using Biplane Simpsons method from apical views. Global longitudinal strain (GLS) were measured using the Philips QLAB analysis package (Philips Healthcare, Andover, USA) from 2D apical views. The E/A ratio represents the ratio of peak velocity blood flow from left ventricular relaxation in early diastole (the E wave) to peak velocity flow in late diastole caused by atrial contraction (the A wave). The E/A ratio was used as a surrogate measure of LV diastolic dysfunction. Tissue Doppler imaging were obtained at levels of the lateral mitral annulus for measurements of e’ wave, a’ wave and S wave.

***Heart Rate Variability:*** ECG traces were uploaded to the study database (MedSciNet) and analysed with Medilog Darwin 2 (ver. 2.7.1) and Kubios HRV Premium (ver. 3.3.1) software ^4^. Heart rate variability (HRV) was analysed from at least three 5-minute segments of the recording obtained. Recordings were visually inspected by a trained researcher and artefacts excluded from analyses. A mean of 8.779 seconds ± 8.674 seconds of the initial section of ECG had to be discarded due to poor tracing quality. Four subjects had artefacts mid-stream and the artefactual data was excluded, with the valid data being split into two distinct sections. Seventeen subjects had significant artefactual tracing during the very end of the session. After visually inspected artefact removal, further artefact reduction was ensured by using the Kubios HRV strong filter during analyses ^5^. For each subject, a time-domain, frequency-domain, non-linear and time-varying analyses was performed. During the frequency-domain analyses data was visualised using the FFT spectrum using the Welch’s periodogram, and the filter values were set as being high frequency (from Hz 0.15 to Hz 0.40), low frequency (from Hz 0.04 to Hz 0.15) and very low frequency (from Hz 0 to Hz 0.04). In the time-domain analyses the NN and PNN threshold was set at 50ms.

Non-linear methods within the Kubios software estimates sympathetic and parasympathetic nervous systems activity by calculating standard deviation 1 (SD1) and SD2 respectively from Poincare plots, where SD1 and SD2 are the standard deviations perpendicular to, and along the line-of-identity (RRn=RRn+1). There was a significant difference in mean for both SD1 and SD2 in the UPBEAT standard care arm compared to normal BMI group after adjustment (SD1, -6·34ms; -12·2 to -0·45: SD2, -7·94ms; -14·81 to -1·07). SD1 describes short-term variability which is mainly caused by respiratory sinus arrhythmia (RSA) and is related to the time-domain measure SDSD (standard deviation of successive differences), whereas SD2 describes long-term variability and has been shown to be related to time-domain measures SDNN and SDSD ^4^ providing a robust index of sympathovagal balance.

SNS index in Kubios HRV software is computed based on Mean HR (bpm), Baevsky’s stress index and SD2 (%) and the PNS index is based on Mean RR (ms), RMSSD (ms) and SD1 (%) and scaled with the standard deviations of a normal population ^6^.

***Supplementary References***

1. Daniels SR, Kimball TR, Morrison JA, Khoury P, Meyer RA. Indexing left ventricular mass to account for differences in body size in children and adolescents without cardiovascular disease. Am J Cardiol 1995; 76(10): 699-701.

2. Lang RM, Badano LP, Mor-Avi V, et al. Recommendations for cardiac chamber quantification by echocardiography in adults: an update from the American Society of Echocardiography and the European Association of Cardiovascular Imaging. Eur Heart J Cardiovasc Imaging 2015; 16(3): 233-70.

3. de Simone G, Daniels SR, Devereux RB, et al. Left ventricular mass and body size in normotensive children and adults: assessment of allometric relations and impact of overweight. J Am Coll Cardiol 1992; 20(5): 1251-60.

4. Tarvainen MP et al., Kubios HRV – heart rate variability analysis software. Computer methods and programs in biomedicine, 113 (1), 210-220, 2014.

5. Tarvainen MP et al., An advanced detrending method with application to HRV analysis. IEEE Transactions on Biomedical Engineering, 49 (2), 172-175, 2002.

6. Nunan D, Sandercock GR, Brodie DA. A quantitative systematic review of normal values for short-term heart rate variability in healthy adults. Pacing Clin Electrophysiol. 2010;33(11):1407-1417.


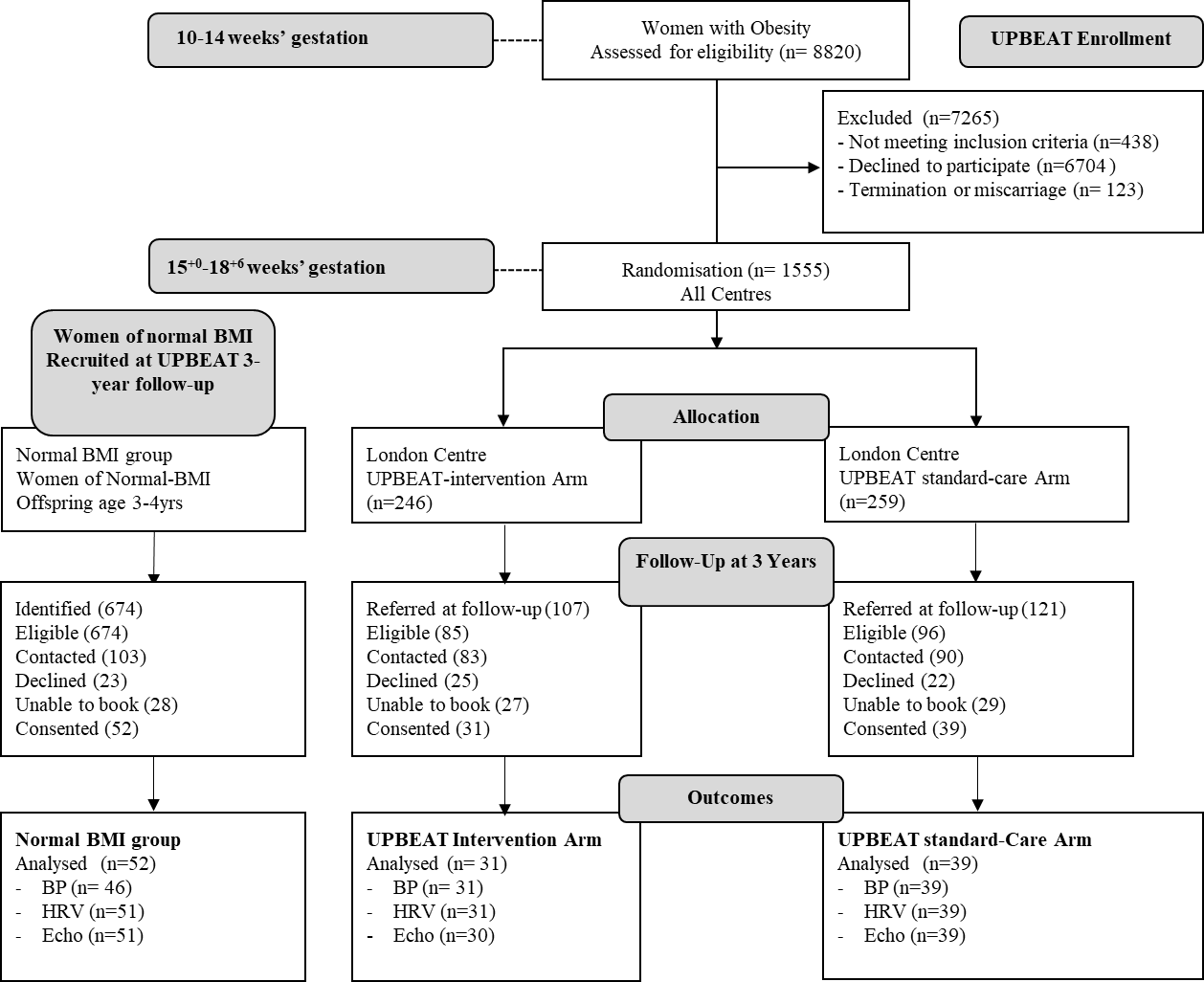


***Supplemental Figure 1: Consort diagram for Recruitment.*** *Mothers and their 3-year-old children attending the UPBEAT RCT follow up visit were informed about the additional nested case control cardiovascular study and invited to return within 2 weeks, when the mother provided consent*

| **Supplemental Table 1:** UPBEAT 3-year follow-up: Comparison of maternal characteristics of those who attended the 3-year follow-up versus those who did not, by randomisation arm. | | | | | | | | | | | | |
| --- | --- | --- | --- | --- | --- | --- | --- | --- | --- | --- | --- | --- |
| **Maternal** | | **UPBEAT main cohort** | | | | | **UPBEAT SUBGROUP** | | | | | **UPBEAT vs UPBEAT SUB-GROUP** |
|  |  | **Intervention** | | **Standard Care** | | **Difference in means (95%CI)** | **Intervention** | | **Standard Care** | | **Differences in means (95%CI)** |  |
|  |  | **Mean (SD)/ Median (IQR) N (%)** | | | |  | **Mean (SD)/ Median (IQR) N (%)** | | | |  |  |
| **Age (years) at baseline** | | 763 | 30·5 ± 5·5 | 758 | 30·4 ± 5·5 | 0·07 (-0·62 to 0·48) | 31 | 31·2 ± 5·4 | 39 | 32·8 ± 5·2 | -1·57 (-4·13 to 0·98) | 0·001 |
| **BMI (kg/m^2^) at baseline** | | 763 | 36·3 ± 4·93 | 758 | 36·3 ± 4·60 | -0·04 (-0·52 to 0·43) | 31 | 36·8 ± 4·2 | 39 | 36·2 ± 5·7 | 0·58 (-1·88 to 3·04) | 0·78 |
| **ethnicity** | Asian | 763 | 43 (6) | 758 | 48 (6) | 0·12 (-0·31 to 0·55) | 31 | 1 (3) | 39 | 1 (2) | 0·87 (0·05 to 15·3) | 0·003 |
|  | Black |  | 194 (25) |  | 194 (26) | 0·01 (-0·22 to 0·24) |  | 10 (36) |  | 19 (48) | 1·66 (0·58 to 4·74) |  |
|  | White |  | 482 (63) |  | 476 (63) | Ref |  | 14 (42) |  | 16 (44) | Ref |  |
|  | Other |  | 44 (6) |  | 40 (5) | -0·08 (-0·53 to 0·36) |  | 6 (19) |  | 3 (6) | 0·43 (0·09 to 2·08) |  |
| **Multiparous** | | 763 | 436 (57) | 758 | 423 (56) | -0·05 (-0·26 to 0·19) | 31 | 15 (48) | 39 | 22 (65) | -0·59 (-1·62 to 0·45) | 0·61 |
| **Smoking status at baseline** | | 763 | 127 (16) | 758 | 120 (16) | -0·06 (-0·33 to 0·21) | 31 | 1 (3) | 39 | 1 (2) | 0·59 (0·14 to 2·43) | 0·45 |
| **Years in education** | | 763 | 14·7 (2·87) | 758 | 14·7 (2·88) | -0·04 (-0·24 to 0·33) | 31 | 14·9 ± 2·90 | 39 | 15·2±3·23 | 0·33 (-1·14 to 1·79) | 0·51 |
| **Mode of delivery** | LSCS in labour | 763 | 111 (15) | 758 | 138 (18) | 0·21 (-0·07 to 0·50) | 31 | 7 (23) | 39 | 5 (13) | 0·85 (-0·44 to 2·13) | 0·08 |
|  | Operative vaginal |  | 94 (12) |  | 85 (11) | -0·10 (-4·2 to 0·22) |  | 2 (6) |  | 4 (10) | -0·18 (-1·98 to 1·61) |  |
|  | LSCS prelabour |  | 159 (21) |  | 136 (18) | -0·15 (-0·42 to 0·11) |  | 4 (13) |  | 7 (18) | -0·05 (-1·41 to 1·31) |  |
|  | Unassisted |  | 299 (52) |  | 399 (53) | Ref |  | 18 (58) |  | 23 (58) | Ref |  |
| *Abbreviations: BMI, body mass index; CI, confidence intervals; IQR, interquartile range; SD, standard deviation; LSCS, Labour lower section caesarean section* | | | | | | | | | | | | |
